# Book Notices

**Published:** 1878-07-20

**Authors:** 


Page(s) missing in pamphlet form are mentioned elsewhere. Dr. Grigg's report on Gynæcology contains many cases and interesting practical suggestions. Dr. Goldsmith's article on the corn-stalk tent, contains a new and practical idea, and will be read, we doubt not, with interest. Also the paper of Dr. Powell, our senior editor, on the Duties of the True Physician, very appropriate just now when the ethics of the profession is at so low a standard. The article by Dr. Love contains new and practical hints. Dr. Baird's article on neuralgia is a good one.

We have not space for further mention at present, and can only add in general terms that the papers are all creditable, and the volume before us a decided improvement on any that has been issued by the Society for a number of years, especially in its typographical execution. As a Georgian, our State pride is gratified with this beautiful volume of Transactions, yet in regard to the internal machinery and management of the Association, evidences continue to reach us of much dissatisfaction in the ranks of the profession. W.

